# A Modular Diabetic Retinopathy Analysis Framework for Lesion Segmentation and Severity Grading: Cross-Dataset External Validation from APTOS to Messidor-2

**DOI:** 10.3390/diagnostics16213596

**Published:** 2026-10-31

**Authors:** Sofiane Boumeshed, Yazid Cherfa, Assia Cherfa, Noureddine Belkhamsa

**Affiliations:** Laboratoire de Physique et Chimie des Matériaux et Interfaces Avancées (LPCMIA), Département d’Électronique, Faculté des Sciences et Technologies, Université Blida 1, Blida 09000, Algeria; cherfa_yazid@yahoo.fr (Y.C.); assia_bz@yahoo.fr (A.C.); nourbelk@hotmail.com (N.B.)

**Keywords:** diabetic retinopathy, computer-aided diagnosis, lesion segmentation, automated grading, cross-dataset external validation, calibration shift, fundus imaging, deep learning

## Abstract

**Background/Objectives**: High in-domain accuracy of diabetic retinopathy (DR) grading models does not guarantee a similar performance for a new population. We quantify how an APTOS-trained grader degrades on an independent cohort and examine why. **Methods**: A modular framework combines a ResNet-34 U-Net for lesion segmentation (IDRiD) with an EfficientNet-B0 severity grader (APTOS 2019), optionally fused with lesion-derived and frequency features. The grader was evaluated zero-shot on Messidor-2 (n=1744) with bootstrap confidence intervals, four training seeds, referable-DR analysis, a discrimination-versus-calibration decomposition, a paired preprocessing ablation, and a duplicate audit. **Results**: Quadratic weighted kappa (QWK) fell from 0.873 (95% CI 0.831–0.909) on the APTOS test set to 0.455 (0.400–0.507) on Messidor-2; across four seeds the external QWK was 0.410±0.057. Referable-DR sensitivity fell from 92.7% to 35.0% (specificity 93.9% and 96.6%) and the AUC from 0.984 to 0.795, indicating both a loss of discrimination and a calibration shift; recalibrating thresholds with target labels (cross-validated) raised QWK only to 0.583. Omitting CLAHE reduced QWK to 0.326 (paired difference 0.129, 0.089–0.171). Thirty-three APTOS test images duplicated in the training or validation data changed QWK marginally (0.866). Lesion-feature fusion did not significantly improve grading. The segmentation Dice on lesion-containing patches was 0.51–0.68, whereas the per-image Dice was 0.21–0.61. **Conclusions**: In-domain agreement is a poor proxy for external performance; multi-seed reporting, explicit calibration analysis, and preprocessing control are needed before clinical use.

## 1. Introduction

Diabetic retinopathy (DR) is a major cause of preventable visual impairment, and automated screening has become an active area of research [1]. Mass screening relies on the reading of fundus photographs, a time-consuming process that depends on the availability of qualified ophthalmologists, hence the clinical interest in automated computer-aided diagnosis (CAD) systems [1,2,3]. For medical-imaging AI, however, evidence of in-domain accuracy is distinct from evidence of applicability to an external population or acquisition setting; current reporting guidance emphasizes explicit description of data provenance, preprocessing, partitioning, external evaluation, and statistical analysis [4,5]. DR-CAD systems typically combine two building blocks: (1) *segmentation* or localization of the characteristic lesions (microaneurysms—MA; hemorrhages—HE; hard exudates—EX; soft exudates—SE) and (2) *classification* of DR severity, generally on the Five-level International Clinical Diabetic Retinopathy (ICDR) scale (grades 0–4) [6,7,8,9,10].

A CAD system trained and validated on a single dataset (with the same image source, acquisition protocol, and population) often achieves a high in-domain performance, but nothing guarantees that this performance will be preserved for images from a different center, device, or population [3,11,12]. Published external-validation studies quantify this gap in different ways. The closest published comparison to the present work uses the same source–target pair, an APTOS-trained EfficientNet-B0-based model evaluated on the 1744 gradable Messidor-2 images, and reports a QWK decrease from 0.896 to 0.642 even after thresholds are calibrated on the APTOS validation set [13]. Domain-generalization benchmarks and methods for DR grading (GDRBench [3], DRGen [11], and preprints on adversarial-blur augmentation [12] and neuro-symbolic single-domain generalization [14]) were developed because models trained on one source often fail on unseen domains.

This work asks four questions about one source–target pair. (i) How large is the external degradation of an APTOS-trained grader on Messidor-2 once statistical uncertainty, run-to-run (seed) variability and data leakage are quantified? (ii) Is the degradation due to a loss of discrimination or mainly a shift in the decision thresholds (calibration)? (iii) How much of the measured gap is attributable to preprocessing inconsistency measured in a paired experiment? (iv) Do lesion-derived features from a dedicated segmentation module improve the generalization of the grader?

The architectures used here are standard (a U-Net with a ResNet-34 encoder for segmentation and EfficientNet-B0 for grading), and no architectural novelty is claimed. The contributions are as follows: (1) An external validation of an APTOS-trained grader on Messidor-2 (n=1744) with bootstrap confidence intervals, four training seeds, exact intervals for screening metrics, and an audit of duplicated APTOS images; (2) a decomposition of the external failure into discrimination loss (AUC), calibration shift (threshold and cut-point re-estimation, reported both as an upper bound and cross-validated), and a paired ablation of the preprocessing step, together with checks of channel order and input resolution; (3) a controlled test of whether lesion-segmentation features and frequency features fused with the image embedding improve internal or external grading, reported as a negative result; and (4) a segmentation evaluation that goes beyond lesion-containing patches (all patches, whole images, false-positive burden, threshold sensitivity, and four independent retrainings with different seeds and validation splits). The study is framed as external validation of a single source–target pair and not as a broad claim of performance across additional datasets.

## 2. Related Work

### 2.1. Public Datasets for Diabetic Retinopathy

The DR-CAD literature relies on a small number of reference datasets: IDRiD, with pixel-level lesion annotations and a segmentation and grading challenge [7,15]; APTOS 2019 for severity grading [16]; and Messidor/Messidor-2, a historical reference for external evaluation [17,18], complemented by DDR and EyePACS for larger-scale studies [6,9]. Benchmarks such as GDRBench aggregate up to eight fundus datasets to standardize the evaluation of domain generalization [3]. The present study uses APTOS as the source dataset and Messidor-2 as an independent external test set without target-domain adjustment.

### 2.2. Architectures for Segmentation and Grading

For lesion segmentation, U-Net-derived architectures with pretrained ResNet-type encoders remain a standard approach [19,20], and IDRiD serves as the reference benchmark through its challenge [7]. For grading, end-to-end CNNs of the EfficientNet family are widely used [8,21]. The in-domain performance reported on APTOS is high: a dual-branch ResNet50/EfficientNet-B0 network reached QWK 0.930 and accuracy 89.6% [22], and a transfer-learning ensemble reached QWK between 0.954 and 0.967 on APTOS depending on the configuration [9]. Our in-domain QWK (0.873–0.893 depending on the seed) is lower than these values, which were obtained with different splits, augmentation strategies, backbones or ensembles, and are therefore not directly comparable with the compact single-model baseline used here.

### 2.3. Ensembles and Lesion-Aware Grading

Ensembles of CNN backbones (VGG16, ResNet, Inception, EfficientNet) are frequently proposed to improve grading robustness [9,23]; in one study, ensembling improved the QWK by 0.011 to 0.023 over the corresponding single model depending on the dataset [9]. Lesion-aware systems combine a severity classifier with lesion localization; for example, a classification CNN was combined with a YOLOv3-based lesion detector on DDR and APTOS [6]. The present study follows a related idea in a deliberately simple form: image-level statistics derived from lesion segmentation are concatenated with the image embedding, and the effect on internal and external performance is tested statistically.

### 2.4. Frequency-Domain Approaches

Frequency-processing branches (wavelet transform—FFT) have been proposed alongside spatial features, notably for retinal vessel segmentation [24]. In the present work, frequency information is provided only as four radial FFT-band energy fractions in a fusion ablation; no architectural novelty is claimed for it.

### 2.5. Within-Dataset Accuracy Versus External Validation

Many recent studies report very high accuracy inside a single dataset. For example, a CNN trained from scratch on Messidor-1 reported 99.36% accuracy for four-grade classification after augmentation and a 70:10:20 partition of the augmented dataset [25], and a ResNet-101 feature extractor with an SVM and a quadrant-mixing augmentation reported 96.09% (binary) and 92.24% (multiclass) accuracy on Messidor-2 alone [26]. Such within-dataset figures cannot be compared with zero-shot external performance. Studies that do perform evaluations across datasets report substantial degradation: APTOS-to-Messidor-2 QWK decreases from 0.896 to 0.642 with calibrated thresholds [13]. Reporting guidelines (CLAIM, STARD-AI) ask for explicit description of data provenance, partitioning, external test sets and statistical analysis [4,5]; the present work follows this guidance by reporting confidence intervals, seed variability and a duplicate audit for the primary comparison.

## 3. Materials and Methods

### 3.1. Study Design

Figure 1 summarizes the study. Module A segments the four lesion types on IDRiD with one binary ResNet-34 U-Net per lesion. Module B grades severity on APTOS 2019 with an EfficientNet-B0 and is evaluated zero-shot on Messidor-2. The modules interact only through an optional late-fusion ablation: image-level lesion features (area fraction and number of connected components for each of the four lesion types, eight values in total) computed from the segmentation module and four FFT-band energy fractions can be concatenated with the EfficientNet-B0 image embedding. The primary results are those of the image-only grader; the fusion variants are used to test whether lesion information improves generalization. Reporting the modules together does not imply a validated end-to-end lesion-to-grade system. All code was implemented in Python (version 3.13.9; Python Software Foundation, Wilmington, DE, USA) with PyTorch (version 2.13.0+cu126; Meta Platforms, Menlo Park, CA, USA), torchvision (version 0.28.0), OpenCV (version 5.0.0), NumPy (version 2.5.2), SciPy (version 1.18.1), scikit-learn (version 1.9.0) and Matplotlib (version 3.11.1).

### 3.2. Datasets

Table 1 summarizes the datasets and Table 2 gives the grade distributions and image characteristics.

*IDRiD.* The official protocol provides 54 training and 27 test images (4288 × 2848 pixels) with pixel-level masks for MA, HE, EX and SE [7,15]. Not every image carries a mask for every lesion type (Section 3.4).

*APTOS 2019.* We used the publicly downloadable APTOS 2019 images in a pre-partitioned form (files train_1.csv, valid.csv and test.csv) with 2930 training, 366 validation and 366 test images (3662 in total) [16]. We did not create this partition, and the procedure used to create it (stratification, patient grouping) is not documented in the distribution; the partition is therefore treated as image-level, and its grade distributions are reported in Table 2. APTOS images come from multiple clinics and cameras and have 17 distinct native sizes (width 474–4288 px, median 2144 px). The dataset contains no patient identifiers, so patient-level separation cannot be verified; instead, we audited near-duplicate images across partitions (Section 3.6).

*Messidor-2.* Messidor-2 [17,18] was reserved exclusively for external evaluation; no Messidor-2 image was used for training, validation, checkpoint selection, or threshold tuning. We used a preprocessed Kaggle (Google LLC, Mountain View, CA, USA) distribution of Messidor-2 (mariaherrerot/messidor2preprocess) [27], in which images are cropped to remove excess black background and resized to 512×512 pixels; the native Messidor images were acquired with a Topcon TRC NW6 non-mydriatic camera (Topcon Corporation, Tokyo, Japan) at a $45^{\circ }$ field of view and have between 1440×960 and 2304×1536 pixels [17]. Messidor-2 itself is distributed without labels; the reference grades were released by third parties. The label file of this distribution (messidor_data.csv) has exactly the structure of the file released by Google Brain for Messidor-2 (columns image_id, adjudicated_dr_grade, adjudicated_dme and adjudicated_gradable; grades adjudicated by a panel of three retina specialists), on which the reference standard of Krause et al. is based [28]. The Kaggle description of the distribution we used does not document the origin of its labels, so we could not verify that the file is an unmodified copy of that release. It lists 1744 images, all flagged as gradable, whereas the original collection contains 1748 images [18]; the difference in four images is consistent with four images having been adjudicated as ungradable in the original release. We applied no additional exclusion. We use the adjudicated five-level ICDR DR grade as the reference standard. Because the grading protocols of APTOS and Messidor-2 differ, label-definition differences are a possible contributor to cross-dataset disagreement that our design cannot separate from image-domain effects.

### 3.3. Preprocessing

Contrast Limited Adaptive Histogram Equalization (CLAHE) was applied identically to APTOS and Messidor-2 images before the grader: conversion to LAB color space, CLAHE on the L channel (clip limit =2.0, tile grid size =8×8) [29], conversion back, a median blur (kernel size 3), and a Gaussian blur (σ=1.0), followed by resizing to 224×224 pixels and scaling to [0,1]. Images are read with OpenCV and are therefore kept in BGR channel order for both datasets and for training and testing; no conversion to RGB and no ImageNet mean/standard-deviation normalization is applied. This departs from the conventions of the ImageNet-pretrained backbone but is identical across all datasets and all experiments, so it cannot explain the cross-dataset difference by itself; Section 4.5 reports a channel-swap control. Messidor-2 images of the Kaggle distribution are supplied already cropped, a step limited to removing excess black background, with no contrast enhancement. An earlier iteration of the pipeline skipped CLAHE for Messidor-2; that condition is re-evaluated here in a paired experiment using the same checkpoint (Section 4.4). The segmentation module uses its own preprocessing (CLAHE as above, median blur of size 3 and Gaussian blur with σ=0.5, on native-resolution images).

### 3.4. Module A: Lesion Segmentation

Each lesion type is segmented by an independent binary model with a ResNet-34-based U-Net (ImageNet-pretrained encoder, decoder with skip connections, no frequency branch; “NoFreq” variant). Images are processed as non-overlapping 128×128 patches at native resolution (726 patches per image). For training, all lesion-containing patches are kept together with empty patches at a 2:1 ratio; validation and test patches are not filtered. Training used Adam (learning rate $10^{-4}$), batch size 64, up to 60 epochs with a step schedule (learning rate halved at 50% and 75% of the epochs), a combined Dice and binary cross-entropy loss, horizontal and vertical flips as the only augmentation, and early stopping on the validation soft Dice (with a patience of eight epochs for MA and five for the other lesions). The binarization threshold was selected on the validation images from the grid {0.5, 0.4, 0.3, 0.25, 0.2, 0.15, 0.1} and fixed for the test set (MA 0.40, HE 0.30, EX 0.40, SE 0.50). Models were trained on an NVIDIA GeForce GTX 1070 GPU (NVIDIA Corporation, Santa Clara, CA, USA) with 23 GB of system RAM.

The 54 official IDRiD training images, sorted by file name, were split by a fixed file-order rule into the first 46 (training) and the last eight (validation; IDs 47–54). This rule is deterministic but not random, and eight images are few for early stopping and threshold selection. We therefore quantified its influence by four additional independent retrainings per lesion (two seeds, each with the fixed validation set and with a random draw of eight validation images; Section 4.7). The official 27 test images were held out throughout. Not every image carries a mask for every lesion type: MA and EX masks exist for 46/46 training and 27/27 test images, HE masks for 45/46 training and 27/27 test images, and SE masks for only 18/46 training and 14/27 test images (all eight validation images have SE masks). The resulting number of test patches containing lesions is 1116 (MA), 1218 (HE), 2165 (EX) and 153 (SE).

*Segmentation metrics.* The primary metric follows earlier work: the mean per-patch Dice, Dice=2TP/(2TP+FP+FN) (with $\varepsilon =10^{-6}$ in numerator and denominator), over test patches that contain lesion pixels, with a 95% confidence interval from a bootstrap over test images (2000 resamples). Because this metric ignores false positives on lesion-free patches, we also report (i) the per-image Dice, computed by pooling the pixels of all patches of an image (negative patches included) and averaged over annotated images, (ii) the fraction of lesion-free patches with at least one predicted pixel, (iii) pooled pixel precision and recall, and (iv) the Dice as a function of the threshold. Including empty patches in a patch-averaged Dice would be dominated by patches on which nothing is predicted and nothing is annotated, and is therefore not used as a headline metric.

### 3.5. Module B: Severity Grading

The grader is an EfficientNet-B0 backbone pretrained on ImageNet, with the classification layer replaced by a five-class linear layer, operating on the preprocessed fundus image at 224×224 resolution [21]. Training used Adam (learning rate $10^{-4}$), cosine-annealing schedule, class-weighted cross-entropy (inverse class frequency), a batch size of 32, and up to 40 epochs with early stopping (with a patience of eight epochs on validation QWK), and the checkpoint with the best validation QWK. Augmentation consisted of horizontal and vertical flips, rotation by up to $\pm 20^{\circ }$, and brightness scaling in [0.9,1.1], each applied with a probability of 0.5. The primary model was trained with seed 42 (best validation QWK 0.912); three further models were trained with seeds 123, 456 and 789 and identical settings. Training used APTOS exclusively; evaluation was performed in-domain (APTOS test) and zero-shot on Messidor-2.

*Fusion variants.* The same EfficientNet-B0 embedding (1280 values) is concatenated with a 32-dimensional embedding (two-layer MLP) of (a) the eight lesion features, (b) four FFT-band energy fractions (the fraction of spectral energy in four equal-width radial bands of the shifted Fourier magnitude of the 256×256 grayscale image), or (c) both. Lesion features are computed with the four segmentation models applied by a sliding window on the whole preprocessed fundus image (with probabilities merged by the pixel-wise maximum, and thresholds as described above): for each lesion type, the area fraction of the binary mask and its number of connected components. Each variant was trained with the same recipe as the image-only grader. Fusion variants are compared with the image-only variant trained in the same session (which is a separate training run from the primary model) by a paired bootstrap of the QWK difference on identical test images (2000 resamples), with Bonferroni-adjusted intervals across the six comparisons (three variants and two test sets).

### 3.6. Evaluation Metrics and Statistical Analysis

Because DR severity is ordinal, the primary grading metric is the quadratic weighted kappa (QWK):

(1) $$\mathrm{QWK}=1-\frac{\sum_{i=0}^{K-1}\sum_{j=0}^{K-1}w_{ij}O_{ij}}{\sum_{i=0}^{K-1}\sum_{j=0}^{K-1}w_{ij}E_{ij}},$$

where K=5, $O_{ij}$ and $E_{ij}$ are the observed and expected agreement matrices, and $w_{ij}={\left(i-j\right)}^{2}/{\left(K-1\right)}^{2}$ [30]. We also report accuracy, macro- and weighted F1, and per-class precision and recall. For referable DR (grade ≥2 versus 0–1), we report sensitivity, specificity, positive and negative predictive values with exact (Clopper–Pearson) 95% intervals, and the area under the receiver-operating-characteristic curve (AUC) computed from the summed softmax probability of grades 2–4, with a bootstrap interval (2000 resamples). Confidence intervals of QWK are percentile intervals from 10,000 bootstrap resamples of the test images; the interval of the difference between the external and in-domain QWK resamples the two test sets independently; the effect of preprocessing is evaluated by a paired bootstrap over identical images. Bootstrap resampling treats images as independent; Messidor-2 contains two images per examination and APTOS has no patient identifiers, so intervals may be slightly optimistic.

*Discrimination versus calibration.* To separate loss of discrimination from displacement of decision boundaries, we (i) report the AUC, (ii) re-estimate the four cut-points applied to the expected grade $\sum_{k}k\;p_{k}$ so as to maximize the QWK, and (iii) re-estimate the threshold on the referable probability that maximizes Youden’s index. Each re-estimation is reported in two forms: fitted and evaluated on the same set (an optimistic upper bound), and estimated by stratified five-fold cross-validation within the set (fit on four folds, applied to the remaining fold), which uses target labels and is therefore not zero-shot.

*Duplicate audit.* Each image was converted to a 96×96 grayscale thumbnail from which a Gaussian-blurred copy (σ=6) was subtracted, and normalized correlation was computed between images of different partitions (horizontally flipped copies included). Pairs with correlation ≥0.98 were regarded as duplicates. APTOS test images that contained such a copy in the training or validation partitions, or an earlier copy in the test partition, were removed to obtain a de-duplicated test subset; pairs with correlation between 0.90 and 0.98 were flagged but not removed. The audit is automatic and was not verified by manual inspection of every pair.

*Seeds.* Means and standard deviations (with n−1 in the denominator) over the four seeds are reported for the image-only grader.

## 4. Results

### 4.1. Grading: In-Domain Versus Cross-Dataset Performance

Table 3 reports the primary results for the image-only grader (seed 42). QWK decreased from 0.873 (95% CI 0.831–0.909) on the APTOS test set to 0.455 (0.400–0.507) on Messidor-2 (Figure 2a), i.e., ΔQWK =−0.417 (95% CI −0.483 to −0.353; relative decrease 47.8%). The macro-F1 fell from 0.639 to 0.343. The per-grade recall on Messidor-2 was 0.96 (grade 0), 0.01 (grade 1), 0.19 (grade 2), 0.08 (grade 3) and 0.54 (grade 4), compared with 0.98, 0.53, 0.83, 0.41 and 0.39 on APTOS (confusion matrices in Figure 3; raw counts, row sums equal the number of images per true grade). On Messidor-2, 1507 of 1744 images (86.4%) were assigned to grade 0, although only 58.3% of images have grade 0 in the reference standard (Figure 2c), and only four of 270 grade 1 images were recognized. In-domain agreement is also driven mainly by grades 0 and 2: recalls for grades 3 and 4 on APTOS were 7/17 and 13/33.

#### Seed Variability

The external estimate depends strongly on the training seed (Table 4). Across the four seeds, the APTOS QWK was 0.882±0.009 and the Messidor-2 QWK 0.410±0.057 (range 0.327–0.455), so that ΔQWK =−0.472±0.065. The primary run (seed 42) is the most favorable of the four on Messidor-2. The referable-DR sensitivity on Messidor-2 ranged from 16.0% to 35.0% (mean 27.7±8.2%). The in-domain QWK is stable across seeds (±0.009), whereas the external QWK varies six-fold more, so that in-domain results provide poor guidance for external stability. A separately trained image-only variant of the fusion ablation (with the same recipe and seed but during a different training session) gave 0.876 and 0.442, consistent with this run-to-run variation.

### 4.2. Referable-DR Detection and Discrimination

Using grades ≥2 as the referable class (Table 5), the sensitivity on APTOS was 92.7% (127/137; 95% CI 87.0–96.4%) with a specificity of 93.9% (215/229; 90.0–96.6%), whereas on Messidor-2 the sensitivity was 35.0% (160/457; 30.6–39.6%) at a specificity of 96.6% (1243/1287; 95.4–97.5%). The external degradation of case detection is therefore large, with a small change in specificity: the model misses roughly two of three referable cases at its default operating point. The AUC for referable DR decreased from 0.984 (0.974–0.993) to 0.795 (0.769–0.819), so that the ranking of images by referable probability degraded, instead of just the operating point (Figure 2b). The AUC for any DR (grade ≥1) decreased from 0.997 to 0.734 (0.710–0.756).

### 4.3. Discrimination Loss Versus Calibration Shift

The error pattern (86.4% of Messidor-2 images predicted as grade 0) suggests a displaced decision boundary, but the AUC shows that discrimination is also reduced. Table 6 separates the two effects. On APTOS, re-estimating cut-points has little effect on the QWK (0.873 to 0.884 cross-validated; 0.902 upper bound). On Messidor-2, the optimal referable threshold on the summed probability of grades 2–4 was 0.007 (versus 0.187 on APTOS), i.e., the network assigns very small referable probabilities to Messidor-2 images that are nevertheless ranked informatively; using such a target-derived threshold in cross-validation results in a sensitivity of 67.6% at a specificity of 77.7%, and re-estimating the grade 4 cut-points raises the QWK from 0.455 to 0.583 (upper bound 0.598). Thus, a substantial part of the external QWK loss (about one third of ΔQWK) can be recovered via recalibration with target labels, but the recalibrated QWK remains far below the in-domain value, consistent with a genuine loss of discrimination (AUC 0.795). Recalibration requires labeled target data and is not a zero-shot property.

### 4.4. Preprocessing Sensitivity: Paired CLAHE Ablation

An earlier iteration of the pipeline had skipped CLAHE for Messidor-2. We reproduced this condition with the same checkpoint by changing only this step at inference (Table 7). Without CLAHE, the Messidor-2 QWK was 0.326 (0.271–0.378) and the referable-DR sensitivity 21.9% (18.2–26.0%); harmonizing the preprocessing improved the QWK by 0.129 (paired bootstrap 95% CI 0.089–0.171), the accuracy from 58.0% to 61.2%, and the AUC from 0.750 to 0.795. This paired experiment replaces the earlier undocumented comparison (approximately 0.33 versus 0.455) and shows that preprocessing inconsistency alone can move the external estimate by more than a quarter of the in-domain-to-external gap.

### 4.5. Channel Order, Duplicates and Dataset Differences

*Channel order:* Both datasets are read with OpenCV and enter the network in the same BGR order during training and testing. The blue appearance of the fundus images in an earlier version of the qualitative figures is consistent with BGR arrays displayed as RGB; the network input order is identical for all datasets (Section 3.3), and the qualitative figures were regenerated (Section 4.8). As a control, swapping the red and blue channels at inference reduced the QWK from 0.873 to 0.700 on APTOS and from 0.455 to 0.074 on Messidor-2, showing that the network relies on color statistics and is sensitive to channel order. The order is identical for all data used here. This does not explain the external gap, but it illustrates the fragility of the pipeline to colour handling.

*Duplicates:* The audit identified 29 APTOS test images with a near-identical copy in the training set and three with a copy in the validation set (in 10 of these pairs the copy carries a different grade), 30 validation images, with a copy in the training set, and 221 duplicate pairs within the training set (52 with different grades), which is consistent with known label noise in APTOS. No APTOS test image had a near-identical counterpart in Messidor-2. Removing the 31 leaked test images and two internal test duplicates (33 in total) left 333 images with QWK 0.866 (0.821–0.908), sensitivity 92.1%, specificity 93.6% and AUC 0.982 (Table 3); the external gap on this subset is −0.411 (95% CI −0.478 to −0.343). Over the four seeds, the de-duplicated APTOS QWK was 0.880±0.014 and the gap −0.470±0.070. Leakage therefore had a small effect on the in-domain estimate and did not explain the external degradation. A looser threshold (correlation ≥0.95, 194 images retained) resulted in QWK 0.860 (0.804–0.908).

*Dataset differences:* Messidor-2 has a different grade distribution (grade 1: 15.5% versus 8.2%; grade 4: 2.0% versus 9.0%; Table 2) and uniform 512×512 images, whereas APTOS images have 17 native sizes, a much larger spread of field-of-view fractions and lower sharpness after resizing (Laplacian variance 53.2 versus 83.7). Input resolution alone does not separate the two datasets. At the same 224×224 input, the recall of grade 1 is 53% on APTOS (16/30) but 1.5% (4/270) on Messidor-2. APTOS images are reduced to the network input by factors between about two and 19 (4.7-fold for the most frequent size, 1050×1050, and about 10-fold for the median size), and the native Messidor-2 images (1440×960 to 2304×1536 pixels) by about 6- to 10-fold, in two steps, in the version we used (native to 512×512, and then to 224×224); any details lost in the first step are not available to us. We did not retrain the grader at a higher input resolution, so an effect of resolution on the absolute detection limit of early lesions, in both datasets, cannot be excluded.

### 4.6. Effect of Lesion-Feature Fusion

Table 8 reports the four fusion variants trained in the same session. None of the fusion variants changed the QWK significantly on either test set after Bonferroni correction. On APTOS, the point estimates were slightly higher for all fused variants (+0.013 to +0.016), with intervals containing zero. On Messidor-2, the point estimates were −0.037 (lesion), −0.005 (frequency) and +0.035 (lesion and frequency), with unadjusted intervals excluding zero for the lesion and the combined variants but in opposite directions, and Bonferroni-adjusted intervals containing zero. The direction of the effect is therefore inconsistent, and the differences are of the same order as the seed-to-seed standard deviation of 0.057 (Table 4). Lesion-derived aggregate features from segmenters trained on 4288 × 2848 IDRiD images and applied to 512–4288 px APTOS and Messidor-2 images, plus four spectral coefficients, did not improve the generalization of the grader. A preprint reports that lesion-based symbolic features fused with a vision transformer improved cross-domain accuracy [14]; our simple late fusion did not, which may reflect differences in feature design, backbone and evaluation protocol. This does not exclude benefits of tighter lesion–grade coupling.

### 4.7. Segmentation (Module A, IDRiD)

Table 9 summarizes the segmentation results. On lesion-containing patches, the deployed models reached a Dice of 0.549 (MA), 0.509 (HE), 0.675 (EX) and 0.572 (SE). Four independent retrainings per lesion (two seeds; fixed or random validation images) gave mean Dice values of 0.557±0.009, 0.518±0.007, 0.676±0.003 and 0.608±0.018, respectively; the fixed and random validation splits produced mean values that differed by no more than 0.012 for every lesion (MA 0.561 versus 0.554; HE 0.517 versus 0.519; EX 0.676 versus 0.677; SE 0.601 versus 0.614). The thresholds selected on the eight validation images varied between runs (0.10–0.50), but the test Dice varied little across the threshold grid (range over thresholds 0.1–0.5 on the test set: MA 0.530–0.549, HE 0.493–0.526, EX 0.665–0.675, SE 0.572–0.590), so the choice of validation images and threshold does not drive the reported values. For soft exudates, only 153 test patches from 14 images contained lesions (and only 18 training images were annotated), so the SE Dice has wide uncertainty (95% CI 0.510–0.631 for the deployed model; run-to-run range 0.595–0.632) and should not be interpreted as a precise estimate.

Evaluation restricted to lesion-containing patches overstates practical performance. The per-image Dice, which includes lesion-free patches, was 0.246 (MA), 0.303 (HE), 0.614 (EX) and 0.209 (SE); 24.5% (MA), 40.5% (HE), 33.8% (EX) and 17.7% (SE) of lesion-free patches contained at least one predicted lesion pixel; and the pooled pixel precision was 0.16, 0.27, 0.68 and 0.16 at recalls of 0.68, 0.56, 0.79 and 0.63. The models are therefore reasonably sensitive but produce many false-positive regions on lesion-free tissue, particularly for microaneurysms, hemorrhages and soft exudates. Figure 4 shows one full test image (median per-image Dice) for each lesion type with the ground truth and the prediction.

### 4.8. Qualitative Grading Examples

Figure 5 shows correctly graded APTOS test examples of grades 0, 2 and 4, and Figure 6 shows Messidor-2 images with true grade ≥1 that were predicted as grade 0. Both sets were drawn at random (seed 42) and not selected for clarity. The under-graded Messidor-2 images are visually heterogeneous and often contain subtle abnormalities; they are illustrative error cases and do not establish a causal relationship between an image characteristic and the model failure.

## 5. Discussion

### 5.1. Magnitude and Interpretation of the External-Performance Gap

The zero-shot QWK decreased from 0.873 to 0.455 (ΔQWK =−0.417, 95% CI −0.483 to −0.353), and the external estimate is unstable across training seeds (0.410±0.057, range 0.327–0.455), with the primary run being the most favorable one. A single-run external estimate should therefore be reported with its seed variability, and comparisons of methods based on differences of a few hundredths of QWK on an external set are unreliable without repeated training. The referable-DR analysis shows that the degradation is concentrated in case detection (sensitivity 92.7% to 35.0%) with almost unchanged specificity, i.e., a systematic under-grading. This pattern is consistent with the strong shift in the referable-class probabilities of the network on Messidor-2 (with an optimal threshold of 0.007) and with the low proportion of predicted grades ≥1.

The decomposition shows that the failure has two components. The first is a calibration component: re-fitting decision cut-points with target labels raises the QWK from 0.455 to 0.583 (cross-validated), and a sensitivity of 67.6% becomes reachable at a specificity of 77.7%. The second is a discrimination component: the AUC falls from 0.984 to 0.795 and cannot be repaired by moving thresholds. Recalibration before deployment is therefore necessary but not sufficient, and it requires labeled local data. Because the cut-point analysis is cross-validated within Messidor-2, it is an approximation of what local recalibration could achieve with a few hundred labeled images, not a zero-shot result.

Preprocessing inconsistency alone reduced the QWK by 0.129 (paired 95% CI 0.089–0.171), which is of the same order as the seed-to-seed variability of the external estimate and larger than the effect of any fusion variant. This supports the need to report the preprocessing of source and target data explicitly [4,5]. The remaining explanations (image quality and camera, population and disease-spectrum differences, label definitions, and the more limited capacity or training-data diversity of the source model) were not isolated experimentally and remain hypotheses. Two candidate explanations were examined: leakage between APTOS partitions does not explain the gap (QWK 0.866 after de-duplication), and input resolution does not differ markedly between the datasets at the network input (Section 4.5), although a retraining at higher resolution was not performed.

### 5.2. Comparison with the Literature

Table 10 places the results in the context of recent studies. Only one of the listed studies evaluates an APTOS-trained model on Messidor-2 without training on Messidor-2 images. The peer-reviewed study of Sheng et al. [13] uses the same 1744 Messidor-2 images and an EfficientNet-B0-based architecture and reports an external QWK of 0.642±0.036 with thresholds calibrated on the APTOS validation set, higher than our four-seed mean of 0.410, but it uses a high-resolution branch, an additional ordinal-risk branch, and calibrated thresholds, and it was not designed as a plain baseline; the reported in-domain QWK (0.896) is close to ours (0.873–0.893), so the larger external QWK cannot be attributed to a higher in-domain accuracy alone. Our result of 0.882→0.410 (four seeds) is lower than in that study, and our plain image-only baseline without recalibration is expected to be weaker than the calibrated dual-branch system. Within-dataset studies report much higher values (QWK up to 0.967 and accuracy up to 99.4%) that are not comparable with external performance, and some of these use augmentation before partitioning [25] or heavy augmentation of the single evaluation dataset [26].

Several plausible factors may contribute to the gap and were not isolated experimentally: (1) the capacity of the compact EfficientNet-B0 baseline; (2) the moderate size and low diversity of the APTOS training set (2930 images, containing many duplicates and label conflicts); and (3) the strict zero-shot protocol, without target-domain adaptation, recalibration, or high-resolution inputs, which differs from studies that calibrate thresholds or perform evaluations within a single dataset. Differences in reference standards (adjudicated grades in Messidor-2 versus the APTOS labeling process) may also contribute.

### 5.3. Lesion Information and Frequency Features

Fusing image-level lesion statistics and spectral energies with the image embedding did not yield a reliable improvement in either domain. The results should be read as a negative finding for this specific, simple late-fusion design rather than as evidence that lesion information is uninformative. Three aspects limit the inference: the segmentation models were trained on 4288 × 2848 IDRiD images and applied to images of very different native size, the lesion features are global aggregates (area and component counts) rather than spatial maps, and the segmentation module itself produces many false positives on lesion-free tissue (Section 4.7). Segmentation performance should not be read as whole-image lesion detection: the lesion-patch Dice (0.51–0.68) is substantially higher than the per-image Dice (0.21–0.61), and the SE results rely on 18 training and 14 test images.

### 5.4. Limitations

This study has several limitations. First, it examines a single source–target pair and a single compact architecture; the findings should not be generalized to other datasets or to ensembles and larger models without additional validation. Second, APTOS and Messidor-2 differ in population, acquisition conditions, resolution, dataset construction and possibly grading protocol, so the residual QWK gap cannot be assigned to a specific source of dataset shift; the Messidor-2 label file could not be traced to its original release with certainty (Section 3.2). Third, the APTOS partition was obtained pre-made without documentation; APTOS has no patient identifiers, our duplicate audit is automatic and correlation-based, and the model was trained with the duplicated images present. Fourth, confidence intervals treat images as independent, although Messidor-2 contains two images per examination. Fifth, no fine-tuning, target-domain adaptation or higher-resolution retraining was tested; the recalibration analyses use target labels and are cross-validated within Messidor-2. Sixth, the pipeline feeds BGR arrays without ImageNet normalization to an ImageNet-pretrained backbone; this is applied consistently but is not the standard convention, and the channel-swap control indicates sensitivity to color handling. Seventh, the segmentation module was evaluated only on IDRiD (never on APTOS or Messidor-2, which have no lesion masks); its file-order validation split contains eight images, and SE annotations are scarce. Finally, all analyses are retrospective.

### 5.5. Practical Implications

Consistent with medical-imaging AI reporting guidance, these results should be interpreted as retrospective cross-dataset external validation rather than as a form of clinical validation [4,5]. An APTOS-trained model should not be assumed to retain its in-domain performance on a new population: with the default decision rule, sensitivity for referable DR fell to 35.0% (mean 27.7% over four seeds) on Messidor-2, and recalibration with local labeled data recovered only part of the loss. Before clinical use on a new target population, independent validation with local data, an explicit calibration analysis, seed-to-seed variability, and preprocessing control should be performed, and the operating point should be chosen on local data.

## 6. Conclusions

This study evaluates a modular DR analysis framework in which lesion segmentation and severity grading are trained separately and can be combined by late feature fusion. The APTOS-trained EfficientNet-B0 grader achieved QWK 0.873 (95% CI 0.831–0.909) in-domain and 0.455 (0.400–0.507) on zero-shot Messidor-2 (0.882±0.009 versus 0.410±0.057 over four seeds). Referable-DR sensitivity fell from 92.7% to 35.0% and the AUC from 0.984 to 0.795, indicating a loss of discrimination in addition to a calibration shift; recalibrating with target labels raised the QWK only to 0.583. Omitting CLAHE reduced the external QWK to 0.326 in a paired experiment, duplicated APTOS images had little effect, and fusing lesion or frequency features did not improve generalization. Segmentation Dice on lesion-containing patches (0.51–0.68) overstates whole-image performance (per-image Dice 0.21–0.61). The main conclusion is methodological and practical: in-domain performance is not a proxy for performance on a new dataset, and external validation with multi-seed reporting, explicit calibration analysis and harmonized preprocessing is required before considering clinical deployment. This study does not by itself establish clinical effectiveness or safety.

## Figures and Tables

**Figure 1:**
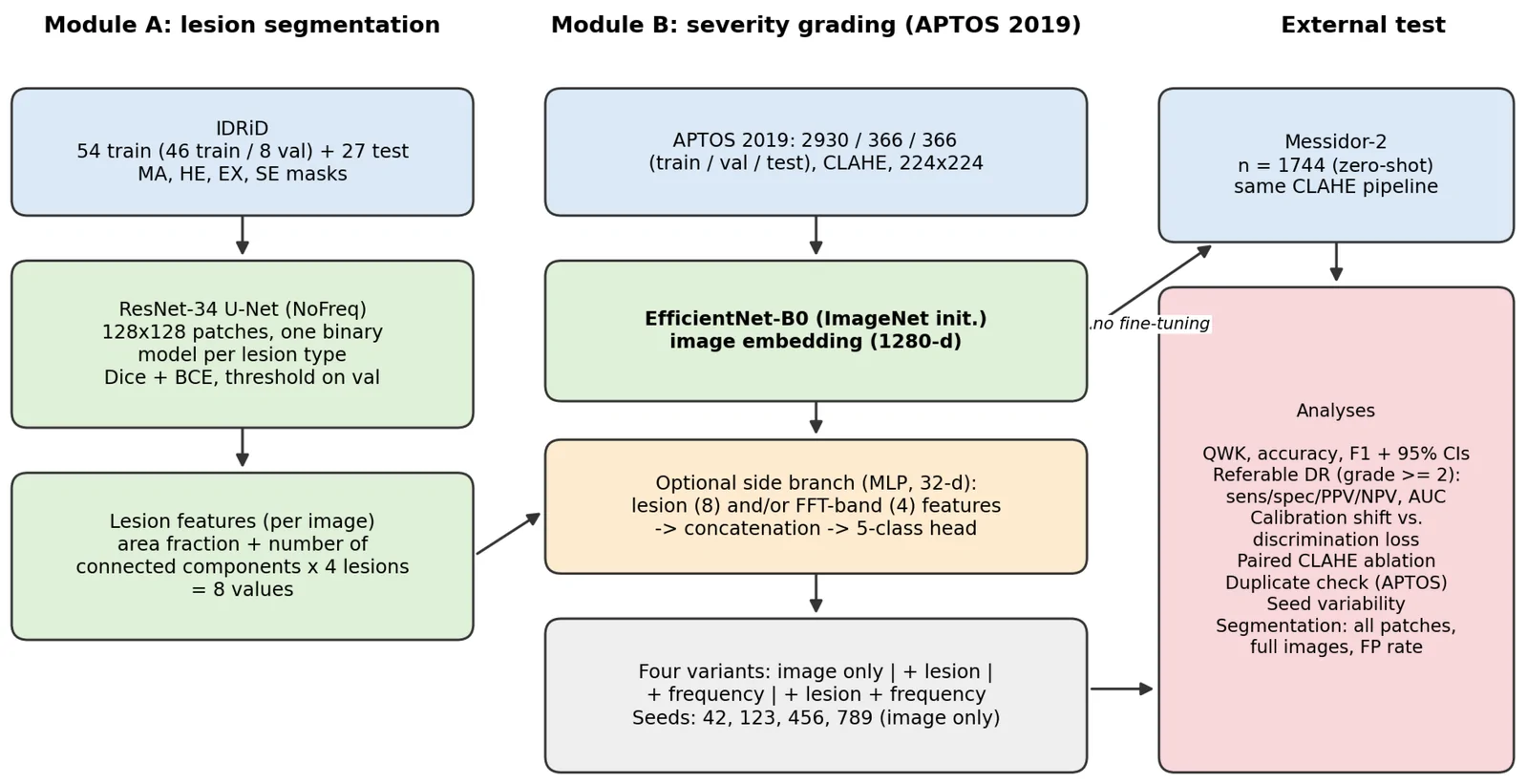
Study design. Module A (lesion segmentation on IDRiD) and Module B (severity grading on APTOS 2019) are trained separately; lesion-derived and frequency features can be fused with the image embedding of the grader. All grading variants are evaluated in-domain (APTOS test) and zero-shot on Messidor-2. The analysis block lists the evaluations reported in this article.

**Figure 2:**
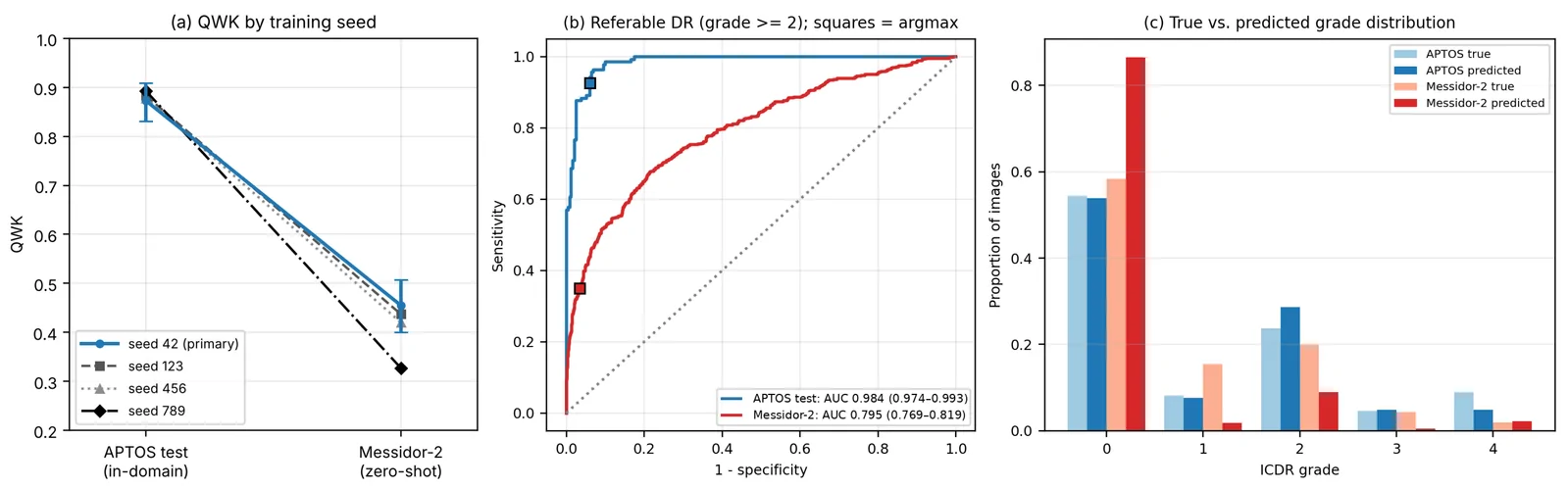
(**a**) QWK of the four independently trained image-only graders (seeds 42, 123, 456 and 789) on the APTOS test set and on Messidor-2; error bars show the 95% bootstrap interval of the primary model (seed 42); the seeds are distinguished by marker and line style. (**b**) Receiver-operating-characteristic curves for referable DR (grade ≥2) of the primary model; squares mark the operating point obtained with the argmax decision rule. (**c**) True and predicted grade distributions of the primary model on both test sets.

**Figure 3:**
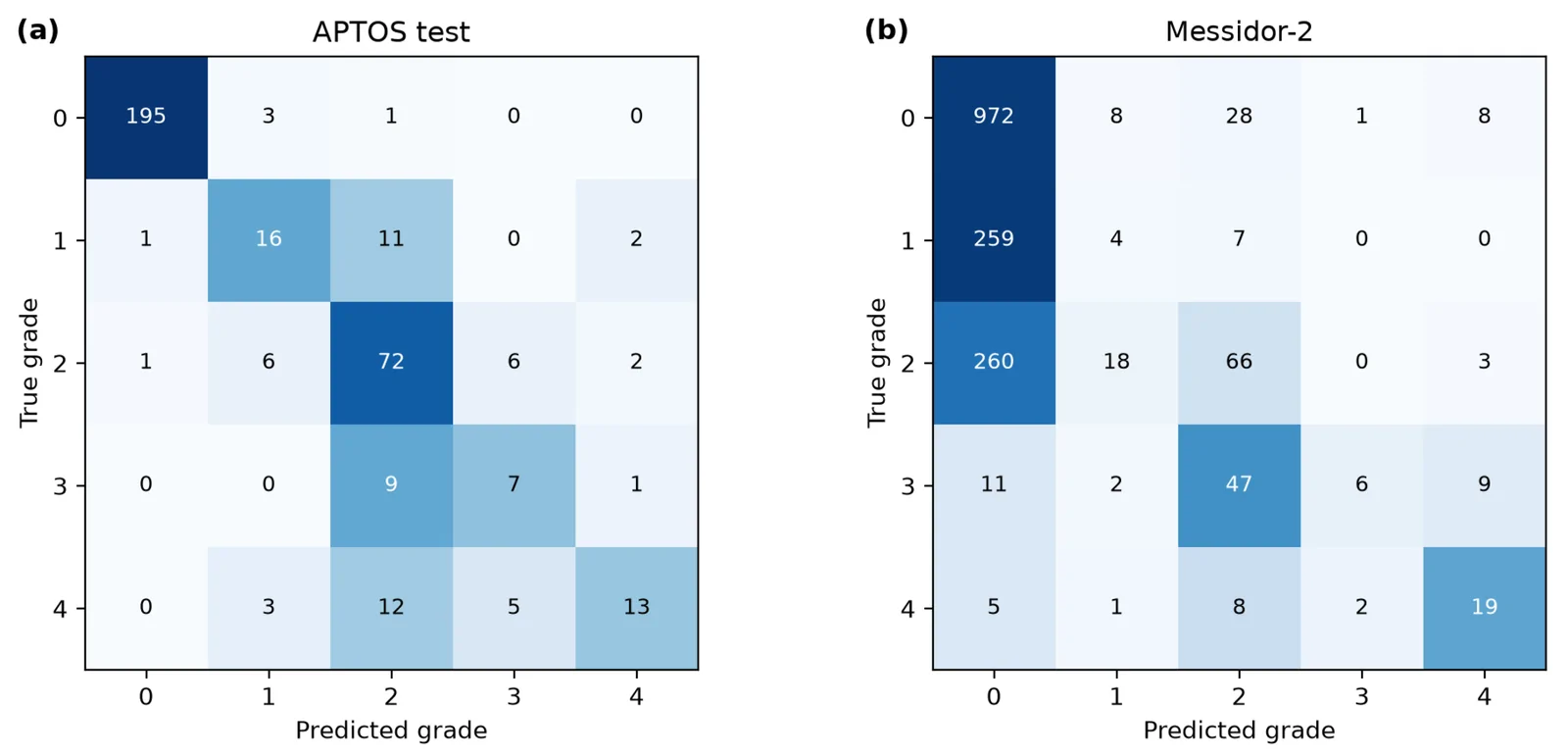
Confusion matrices (number of images) of the primary grader: (**a**) APTOS test (in-domain); (**b**) Messidor-2 (zero-shot). Rows: true grade; columns: predicted grade. Darker shading indicates a larger number of images.

**Figure 4:**
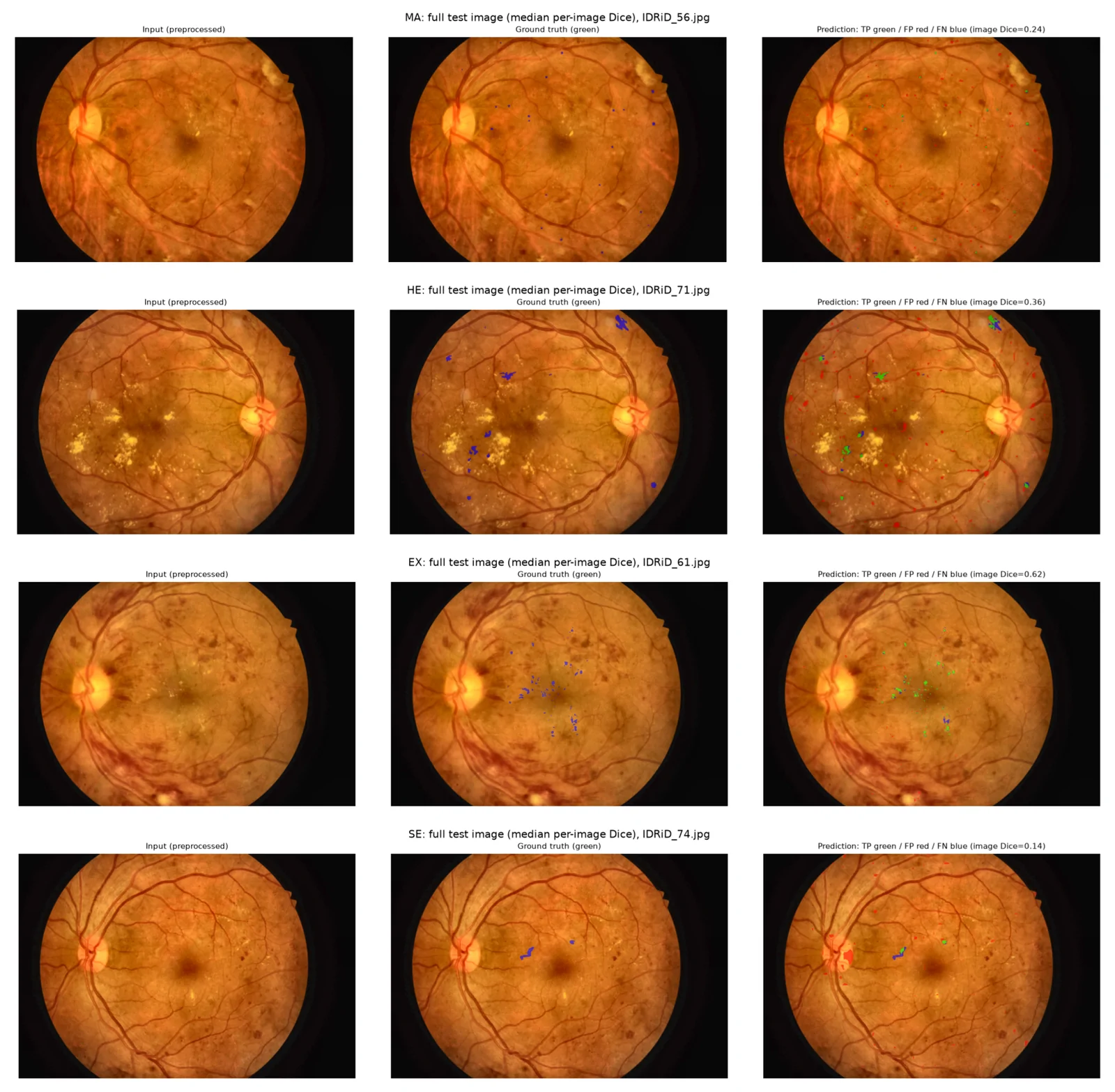
Full IDRiD test images with the median per-image Dice for each lesion type (from top to bottom: MA, HE, EX, SE). (**Left**): preprocessed input; middle: ground truth (green); (**right**): prediction with true positives in green, false positives in red and false negatives in blue. False-positive regions on lesion-free tissue are visible for all lesion types.

**Figure 5:**
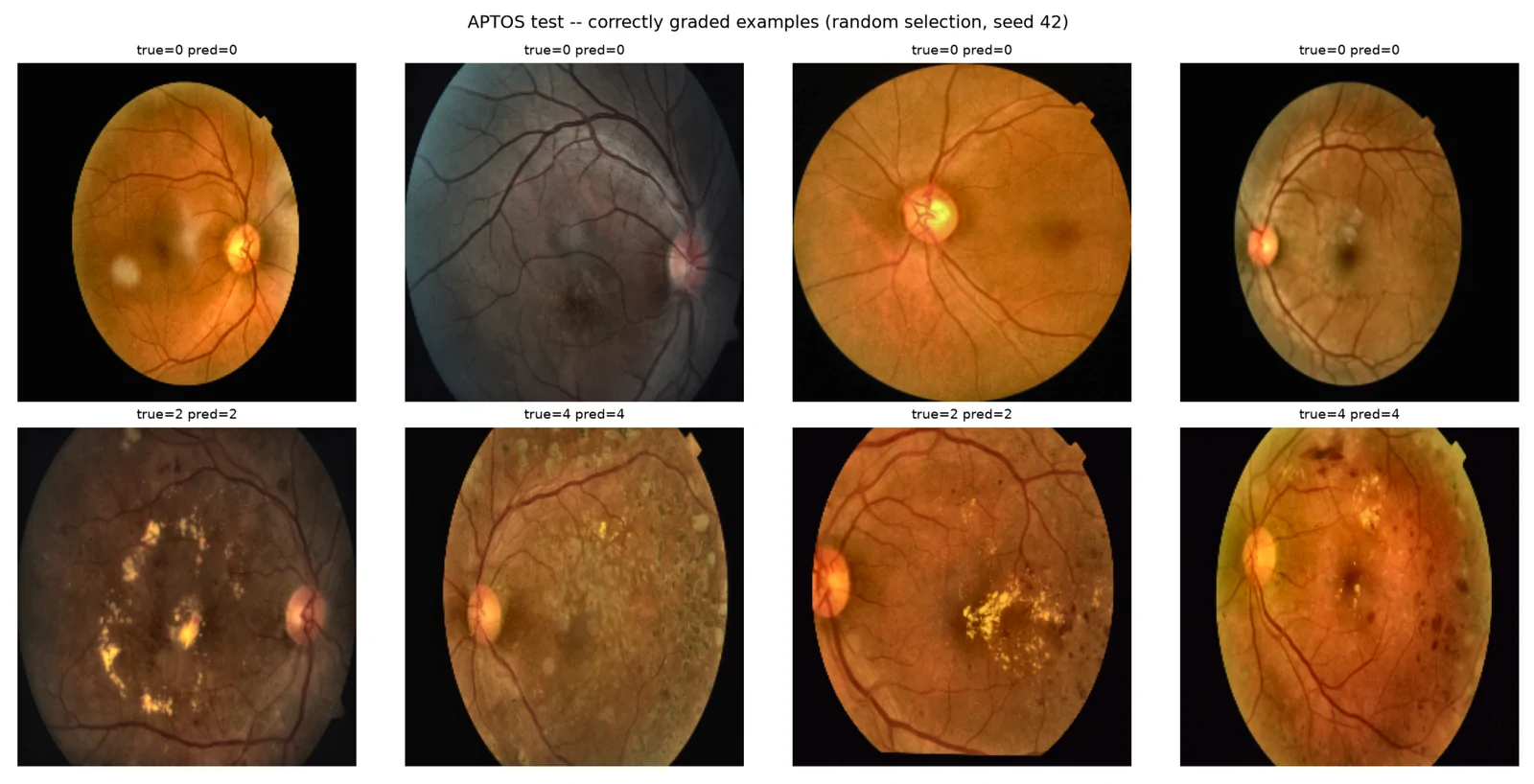
Correctly graded examples, APTOS test set (in-domain), and random selection.

**Figure 6:**
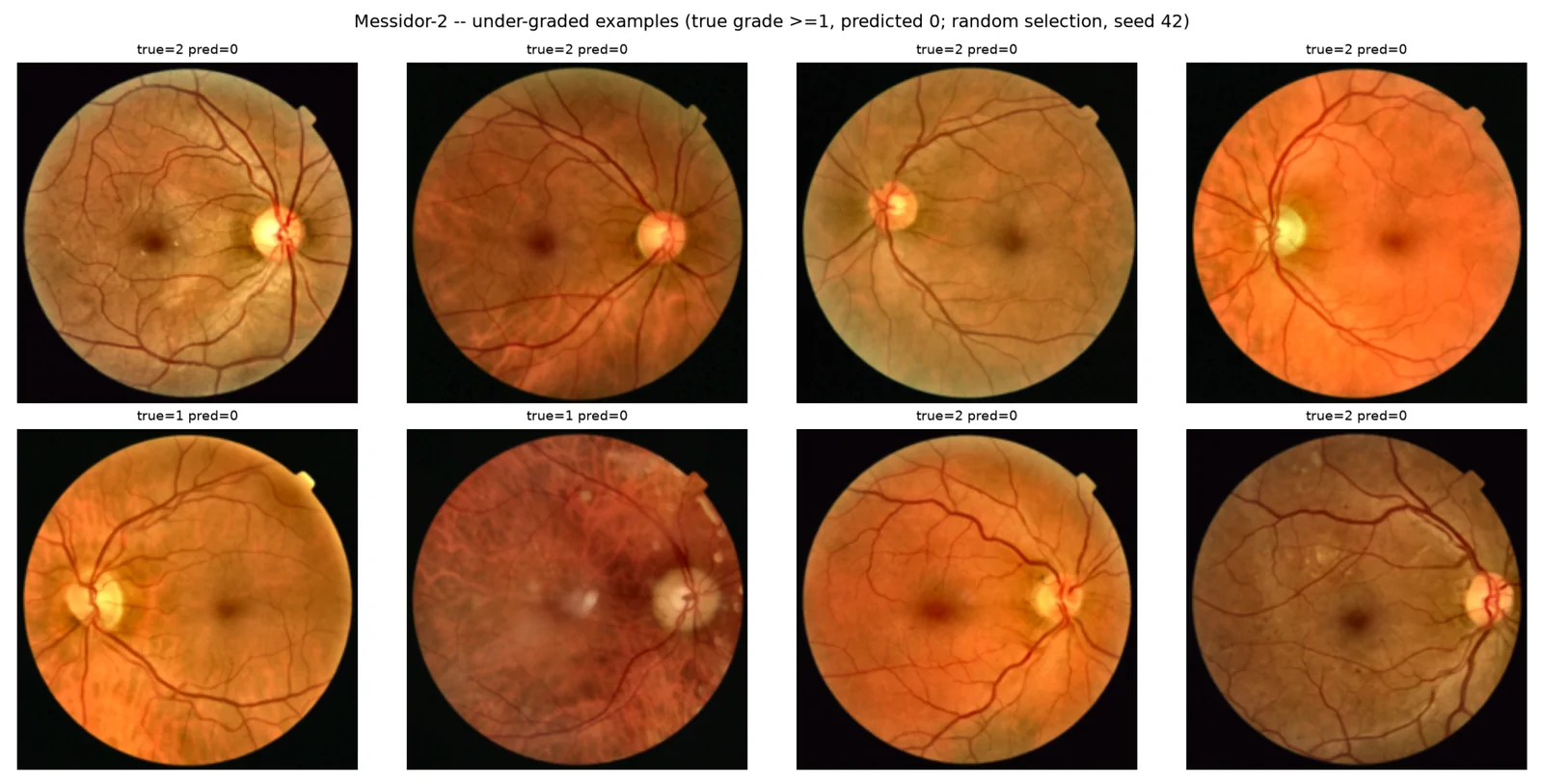
Under-graded examples, Messidor-2 (true grade ≥1, predicted grade 0), and random selection.

**Table 1:** Datasets used in this study.

Dataset	Role	Partition	Task
IDRiD	Training and testing of the segmentation module	46 train/8 validation (from the 54 official training images)/27 test	Segmentation of 4 lesion types (MA, HE, EX, SE)
APTOS 2019	Training, validation and in-domain testing of the grading module	2930/366/366 (train/validation/test)	DR severity, ICDR grades 0–4
Messidor-2	External testing only (zero-shot)	n=1744 (adjudicated, gradable)	DR severity, ICDR grades 0–4

**Table 2:** Grade distributions (number of images and percentage) and image characteristics of the grading datasets. Image characteristics were measured on 150 randomly drawn images per dataset after resizing to 512×512 (mean ± SD): field-of-view fraction, mean gray level, gray-level standard deviation, and variance of the Laplacian (sharpness).

Set	Grade 0	Grade 1	Grade 2	Grade 3	Grade 4
APTOS train (2930)	1434 (48.9%)	300 (10.2%)	808 (27.6%)	154 (5.3%)	234 (8.0%)
APTOS validation (366)	172 (47.0%)	40 (10.9%)	104 (28.4%)	22 (6.0%)	28 (7.7%)
APTOS test (366)	199 (54.4%)	30 (8.2%)	87 (23.8%)	17 (4.6%)	33 (9.0%)
Messidor-2 (1744)	1017 (58.3%)	270 (15.5%)	347 (19.9%)	75 (4.3%)	35 (2.0%)
**Dataset**	**Native size (px)**	**FOV fraction**	**Gray mean/SD**	**Sharpness**	
APTOS 2019	17 sizes; median 2144×1536; most frequent 1050×1050 (974 images)	0.776±0.132	87.7±19.5/ 18.1±6.2	53.2±29.0	
Messidor-2 (Kaggle version)	512×512 (all 1744 images; native images 1440×960 to 2304×1536)	0.742±0.005	93.3±21.5/ 15.1±3.4	83.7±40.9	

**Table 3:** Classification performance of the image-only EfficientNet-B0 grader (seed 42) in the in-domain versus the zero-shot cross-dataset. The 95% confidence intervals are percentile bootstrap intervals (10,000 resamples of the test images). The de-duplicated APTOS test subset excludes images with a near-identical copy in the training/validation partitions or earlier in the test partition (Section 3.6).

Test Set	*n*	QWK (95% CI)	Accuracy	Macro-F1	Weighted F1
APTOS test (in-domain)	366	0.873 (0.831–0.909)	82.8%	0.639	0.824
APTOS test, de-duplicated	333	0.866 (0.821–0.908)	83.5%	–	–
Messidor-2 (zero-shot)	1744	0.455 (0.400–0.507)	61.2%	0.343	0.522

**Table 4:** Seed variability of the image-only grader. Referable DR: grade ≥2. Mean ± SD over the four seeds (SD with n−1).

Seed	APTOS QWK	Messidor-2 QWK	ΔQWK	Messidor-2 Sens.	Messidor-2 Spec.	Messidor-2 AUC
42 (primary)	0.873	0.455	−0.417	35.0%	96.6%	0.795
123	0.883	0.437	−0.446	30.9%	98.0%	0.806
456	0.878	0.420	−0.458	29.1%	98.1%	0.812
789	0.893	0.327	−0.566	16.0%	99.3%	0.750
Mean ± SD	0.882±0.009	0.410±0.057	−0.472±0.065	27.7±8.2%	98.0±1.1%	0.791±0.028

**Table 5:** Referable-DR detection (grades ≥2) of the primary grader with exact 95% confidence intervals (Clopper–Pearson) and AUC (bootstrap).

Test Set	Sensitivity	Specificity	PPV	NPV
APTOS test (TP 127, FN 10, TN 215, FP 14)	92.7% (87.0–96.4)	93.9% (90.0–96.6)	90.1% (83.9–94.5)	95.6% (92.0–97.8)
Messidor-2 (TP 160, FN 297, TN 1243, FP 44)	35.0% (30.6–39.6)	96.6% (95.4–97.5)	78.4% (72.1–83.9)	80.7% (78.7–82.7)
	**AUC, referable DR (grade ≥2)**			
APTOS test	0.984 (0.974–0.993)			
Messidor-2	0.795 (0.769–0.819)			

**Table 6:** Discrimination and recalibration analysis of the primary grader. Cut-points and thresholds are re-estimated on the evaluated set (in-sample, optimistic upper bound) or by stratified five-fold cross-validation within the set (CV); both use target labels.

Test Set	QWK (Default)	QWK, Re-Fitted Cut-Points (In-Sample)	QWK, Re-Fitted Cut-Points (CV)	Referable Threshold (In-Sample)	Sens./Spec. at Re-Fitted Threshold (CV)	Predicted Grade-0 Share
APTOS test	0.873	0.902	0.884	0.187	95.6%/92.1%	53.8%
Messidor-2	0.455	0.598	0.583	0.007	67.6%/77.7%	86.4%

**Table 7:** Paired ablation of CLAHE on Messidor-2 (same checkpoint and images; only the preprocessing step differs).

Preprocessing on Messidor-2	QWK (95% CI)	Accuracy	Referable Sens.	Referable Spec.	AUC (Referable)
CLAHE + blur (as in training)	0.455 (0.400–0.507)	61.2%	35.0%	96.6%	0.795
No CLAHE (cropped images only)	0.326 (0.271–0.378)	58.0%	21.9%	97.1%	0.750
Paired difference	+0.129 (0.089–0.171)				

**Table 8:** QWK of fusion variants and paired bootstrap difference to the image-only variant of the same session (2000 resamples; unadjusted 95% interval and Bonferroni-adjusted interval at level 0.05/6). No difference remained significant after adjustment.

Variant	APTOS QWK	Difference on APTOS [95% CI; Bonferroni CI]	Messidor-2 QWK	Difference on Messidor-2 [95% CI; Bonferroni CI]
Image only	0.876	–	0.442	–
+lesion features	0.889	+0.013 [−0.011, 0.037; −0.020, 0.044]	0.405	−0.037 [−0.070, −0.004; −0.078, 0.004]
+frequency features	0.892	+0.016 [−0.004, 0.037; −0.010, 0.045]	0.437	−0.005 [−0.032, 0.021; −0.041, 0.027]
+lesion + frequency	0.890	+0.014 [−0.019, 0.048; −0.029, 0.066]	0.476	+0.035 [0.003, 0.064; −0.007, 0.073]

**Table 9:** Segmentation performance on the IDRiD test set (ResNet-UNet-NoFreq). Dice: mean per-patch Dice over lesion-containing test patches at the fixed threshold, with 95% bootstrap interval over test images. Four retrainings: mean ± SD [range] of the test Dice of four independent runs (seeds 43 and 44, fixed or random validation set) with their own validation-tuned thresholds. Per-image Dice pools all patches of an image (lesion-free patches included). FP-patches: fraction of lesion-free patches with at least one predicted pixel. Precision and recall: pooled over all test pixels.

Lesion	Annotated Test Images/ Lesion Patches	Threshold	Dice, Lesion Patches (95% CI)	Four Retrainings	Per-Image Dice (95% CI)	FP-Patches	Precision	Recall
MA	27/1116	0.40	0.549 (0.529–0.570)	0.557±0.009 [0.548–0.569]	0.246 (0.213–0.280)	24.5%	0.16	0.68
HE	27/1218	0.30	0.509 (0.465–0.558)	0.518±0.007 [0.512–0.525]	0.303 (0.240–0.365)	40.5%	0.27	0.56
EX	27/2165	0.40	0.675 (0.636–0.703)	0.676±0.003 [0.674–0.680]	0.614 (0.549–0.673)	33.8%	0.68	0.79
SE	14/153	0.50	0.572 (0.510–0.631)	0.608±0.018 [0.595–0.632]	0.209 (0.138–0.292)	17.7%	0.16	0.63

**Table 10:** Comparison with recent studies. “External” means that the target dataset was not used for training. Values are as reported in the cited studies; protocols differ and the values are not directly comparable.

Study (Venue, Year)	Model/Approach	Data and Protocol	Metric, In-Domain	Metric, External
This work (2026)	EfficientNet-B0, image only, 224 px; four seeds	APTOS → Messidor-2 (1744), zero-shot	QWK 0.873 (seed 42); 0.882±0.009 (4 seeds)	QWK 0.455 (seed 42); 0.410±0.057 (4 seeds); 0.583 with CV-recalibrated cut-points
Sheng et al. (Front. Endocrinol., 2026) [13]	Dual-branch EfficientNet-B0 with lesion-order-sensitive risk branch, calibrated thresholds	APTOS → Messidor-2 (1744 gradable); thresholds from APTOS validation	QWK 0.896±0.005; accuracy 0.835; macro-F1 0.683	QWK 0.642±0.036; macro-F1 0.458
Chilukoti et al. (BMC Med. Inform. Decis. Mak., 2024) [9]	Transfer learning and ensembles of EfficientNet models, EyePACS pretraining	Internal splits of EyePACS, APTOS and Messidor-2	QWK 0.901 (EyePACS), 0.967 (APTOS), 0.944 (Messidor-2)	Not evaluated
Shakibania et al. (Biomed. Signal Process. Control, 2024) [22]	Dual-branch ResNet50/ EfficientNet-B0	Multi-center training set including APTOS; APTOS evaluation	QWK 0.930; accuracy 89.6%	Not evaluated
Akhtar et al. (Sci. Rep., 2025) [25]	CNN trained from scratch (RSG-Net)	Messidor-1; augmented data split 70:10:20; four grades	Accuracy 99.36%	Not evaluated
Çelebi et al. (Arab. J. Sci. Eng., 2026) [26]	ResNet-101 features, SVM, quadrant-mixing augmentation	Messidor-2 only; binary and multiclass	Accuracy 96.09% (binary), 92.24% (multiclass)	Not evaluated

## Data Availability

The datasets analyzed in this study are publicly available third-party datasets: IDRiD (https://idrid.grand-challenge.org/ (accessed on 22 August 2026)), APTOS 2019 Blindness Detection (https://www.kaggle.com/c/aptos2019-blindness-detection (accessed on 31 August 2026)), and Messidor and Messidor-2 images (ADCIS, https://www.adcis.net/en/third-party/messidor/ (accessed on 31 August 2026)), with adjudicated grades released on Kaggle by Google Brain (“MESSIDOR-2 DR Grades”); the preprocessed version and label file used here are at https://www.kaggle.com/datasets/mariaherrerot/messidor2preprocess (accessed on 20 September 2026). The code used for preprocessing, model training, evaluation, and figure generation, together with the machine-readable files of all numerical results reported in this article, will be made publicly available in a repository upon publication.
